# Controlling Morphological Parameters of Anodized Titania Nanotubes for Optimized Solar Energy Applications

**DOI:** 10.3390/ma5101890

**Published:** 2012-10-19

**Authors:** Andrew Haring, Amanda Morris, Michael Hu

**Affiliations:** 1 Department of Chemistry, Virginia Tech, Blacksburg, VA 24061-0212, USA; E-Mail: aharing@vt.edu; 2 Energy and Transportation Science Division, Oak Ridge National Laboratory, Oak Ridge, TN 37831, USA

**Keywords:** titania, TiO_2_, nanotube, water oxidation, bulk heterojunction solar cells, dye-sensitized solar cells, photoelectrochemical catalysis

## Abstract

Anodized TiO_2_ nanotubes have received much attention for their use in solar energy applications including water oxidation cells and hybrid solar cells [dye-sensitized solar cells (DSSCs) and bulk heterojuntion solar cells (BHJs)]. High surface area allows for increased dye-adsorption and photon absorption. Titania nanotubes grown by anodization of titanium in fluoride-containing electrolytes are aligned perpendicular to the substrate surface, reducing the electron diffusion path to the external circuit in solar cells. The nanotube morphology can be optimized for the various applications by adjusting the anodization parameters but the optimum crystallinity of the nanotube arrays remains to be realized. In addition to morphology and crystallinity, the method of device fabrication significantly affects photon and electron dynamics and its energy conversion efficiency. This paper provides the state-of-the-art knowledge to achieve experimental tailoring of morphological parameters including nanotube diameter, length, wall thickness, array surface smoothness, and annealing of nanotube arrays.

## 1. Introduction

Ordered TiO_2_ nanostructures, including nanoparticles, nanotubes, and nanorods [1,2] have garnered much research for their use in solar energy applications [3,4,5]. In hybrid solar cells, the titania nanostructures accept electrons from photoexcited dye molecules or polymers adsorbed to the surface and direct the electrons into an external circuit. In photoelectrochemical cells for the degradation of pollutants or the oxidation of water, the photoexcited titania nanostructures donate electrons or holes to chemical species adsorbed to the surface. TiO_2_ nanotubes have also been experimentally applied as gas sensors and supercapacitors but these applications will not be discussed [6,7].

**Figure 1:**
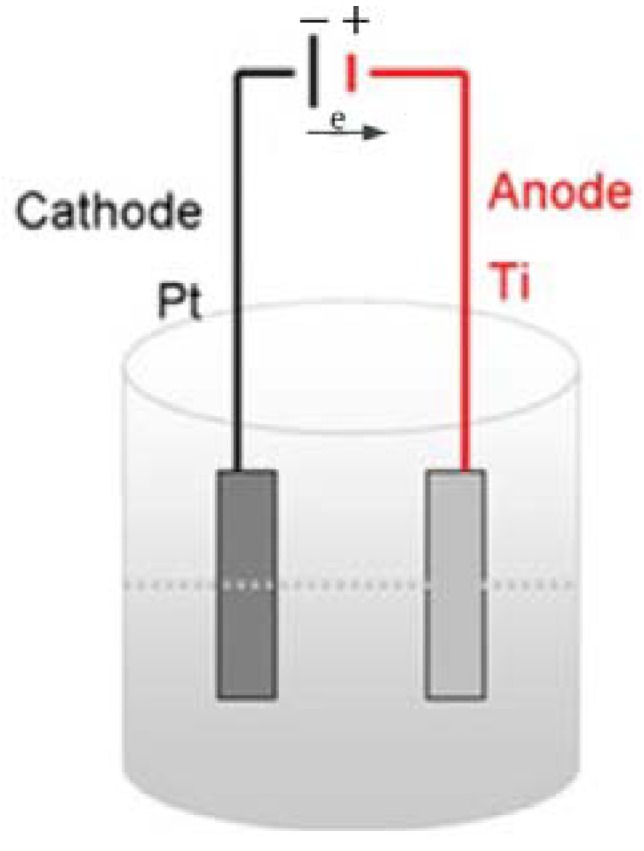
Basic setup for the anodization of titanium to titania nanotubes. Reprinted with permission from [14].

In 1999, Zwilling *et al.* reported on the anodization of titanium in solutions of fluoride-containing electrolytes to form porous titania nanotubes and Gong *et al.* later formed nanotubes using higher voltages (Figure 1) [8,9]. Although titania nanotubes can also be formed by other routes [10], the anodization method leads to an aligned array with an adjustable morphology that can be optimized for its various applications. The morphology parameters, e.g., nanotube length, diameter, smoothness, depend on the anodization conditions, such as voltage, electrolyte composition, temperature, and duration. After anodization, the amorphous nanotubes can be annealed to increase the electron mobility, sensitized with dyes or polymers to increase solar photon absorption, and doped or surface-functionalized to adjust the density of states [11,12,13].

Since Honda and Fujishima reported water oxidation by titania thin films in 1972, titania nanoparticles, nanorods and nanotubes have been investigated [15,16,17,18]. Due to their hollow nature, nanotubes have twice the surface area per unit volume compared to nanoparticles and nanorods that have the same outside diameter as the nanotubes. Recently, Zhang and Wang fabricated a photoelectrochemical cell for water splitting that achieved a photoconversion efficiency of 0.84% under AM 1.5 illumination using titania nanotubes without any catalysts [18].

**Figure 2:**
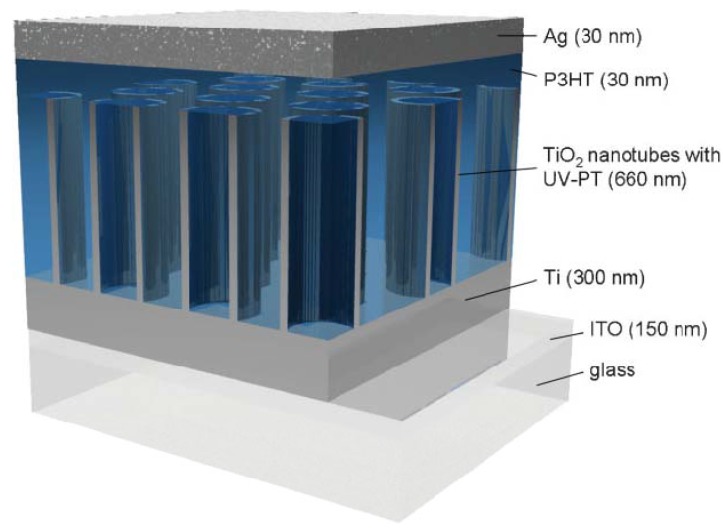
Solid-state solar cell with nanotubes sensitized by polythiophene polymerized in the nanotubes. Reprinted with permission from [21]. Copyright 2009 Wiley.

Hybrid solar cells with titania nanotubes, illustrated in Figure 2, have several advantages over other nanostructures and planar solar cells. Nanotubes, which are aligned perpendicular to the conducting substrate, increase electron mobility within the nanotube by directing electrons along a shorter path than nanoparticles [19,20]. The high surface area of nanotubes, compared to nanorods or flat surfaces, allows for more adsorption by electron donors such as molecular dyes and polymers, thus increasing solar photon absorption and charge collection [21]. Commonly used donors include ruthenium polypyridyl complexes (N719, N749), porphyrin dyes, poly(3-hexylthiophene), and poly(p-phenylene vinylene) derivatives [22,23,24,25]. Although titania nanotubes have attracted extensive research as photoanodes in hybrid solar cells, there are several complications that need to be overcome including phase separation between electron donors and titania, polymer penetration into the nanotubes, and efficient electrical contact with conductive glass [20,26].

## 2. Anodized Titania Nanotube Formation

The formation of titania nanotubes by potentiostatic anodization proceeds by similar mechanisms as porous alumina [27,28]. In the first step of the anodization process, the titanium surface is electrochemically oxidized. A compact layer of titanium oxide is formed on the titanium surface through Equation (1) [27,28,29].

(1) $${\left[{\text{TiF}}_{6}\right]}^{2-}\;+\;2{\text{H}}_{2}\text{O }\to {\text{ TiO}}_{2}\;+\;4{\text{H}}^{+}\;+\;6{\text{F}}^{-}$$

The electrolyte typically contains 0.1 M HF or NH_4_F providing fluoride ions that complex with Ti^4+^, Equation (2), and dissolve TiO_2_, Equation (3) [27,28,29].

(2) $${\text{Ti}}^{4+}+6{\text{F}}^{-}\;\to \;{\left[{\text{TiF}}_{6}\right]}^{2-}$$

(3) $${\text{TiO}}_{2}+\;6{\text{F}}^{-}+4{\text{H}}^{+}\to {\left[{\text{TiF}}_{6}\right]}^{2-}+\;2{\text{H}}_{2}\text{O}$$

**Figure 3:**
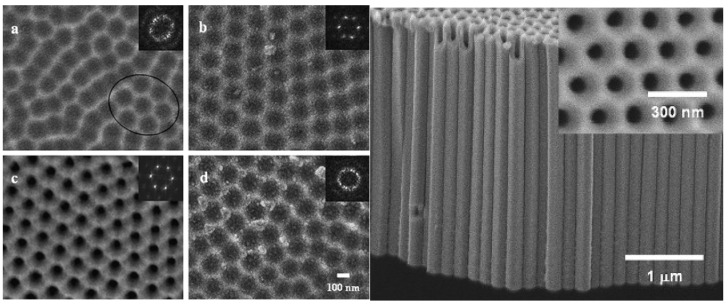
Scanning electron microscopes (SEM) of titanium substrate after removal of (**a**) first; (**b**) second; and (**d**) third nanotube array. Reprinted with permission from [30]. Copyright 2007 Elsevier.

**Figure 4:**
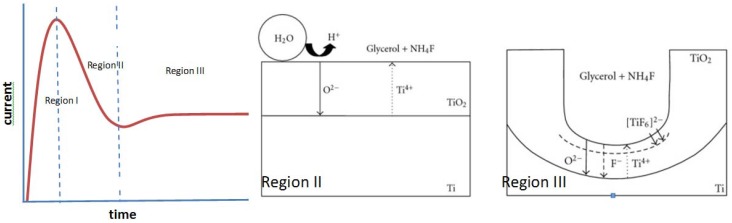
The current profile and nanotube formation scheme are shown. The current increases sharply in Region I as voltage is applied across the electrolyte and bare titanium foil. A compact titanium dioxide layer forms in Region II. In Region III, oxide formation and dissolution reach a steady state and nanotubes form (Equations (1)–(3)). Illustrations reprinted from [31]. Creative Commons 2010.

Pitting of the oxide layer provides preferential locations for the field-assisted chemical dissolution of TiO_2_ by fluoride ions through Equations (2) and (3) [27,29]. Nanotubes are formed as the pits are chemically dissolved further into the oxide layer; the pits provide the least resistive route for the current, therefore the high dissolution rate forms the inside of the tubes from the pits. To form highly ordered nanotubes, the first nanotube array is often removed from the titanium foil leaving indentations that facilitate the pitting behavior during re-anodization (Figure 3) [30]. During the formation, the current typically behaves as illustrated in Figure 4. As the voltage increases to its set magnitude, the current increases (Region I) until the oxide layer provides enough resistance and the current decreases (Region II). The current increases again as Equation (2) begins to increase the surface area and thin the oxide layer. The oxidation continues and a steady state between Equations (1)–(3) is reached in Region III (Figure 4) [3,28]. Wang *et al.* recently published on the formation of metal oxides by anodization and analyze the formation thermodynamically and mechanistically [27].

### 2.1. Control of Morphology

Many experimental conditions of the anodization process are controlled to form nanotubes with the desired morphology: duration, applied voltage, temperature, Ti foil roughness, electrolyte composition. While the duration, voltage, and fluoride concentration primarily controls the nanotube length, diameter, and growth rate, many characteristics of the electrolyte affect the process including the solvent, water content, pH, viscosity, conductivity, and organic additives. Table 1 shows the range of conditions that have been used to form titania nanotubes.

**Table 1:** Range of conditions used to control the nanotube morphology. Electrolyte age refers to its previous use for titanium anodization.

Voltage	10–240V [32,33]	Electrolyte Solvent	water, ethylene glycol, diethylene glycol, DMSO, DMF, formamide, acetic acid [34,35]
Duration	seconds–9 days [36,37]	Water Content	0%–100% [38]
Etching species	HF, NH_4_F, Bu_4_NF [34]	Electrolyte Additives	Na_2_EDTA, H_2_O_2_ lactic acid [31,36,39]
Fluoride Conc.	0.05–0.5M NH_4_F [3,31]	Electrolyte Age	unused-120 h [40]

#### 2.1.1. Nanotube Length

**Figure 5:**
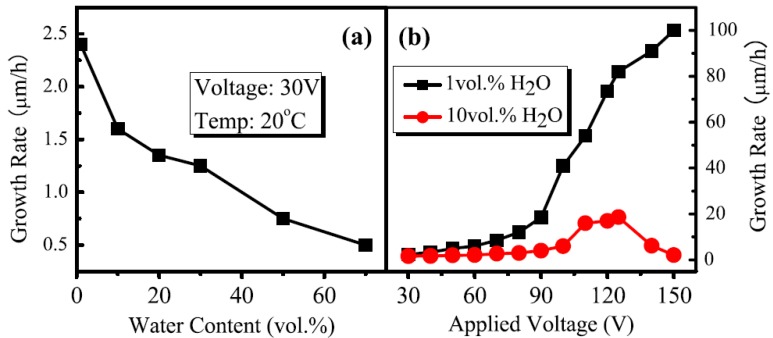
Effect of (**a**) water content and (**b**) anodization voltage on the nanotube growth rate using 0.09 M (0.3 wt %) NH_4_F in ethylene glycol. Reprinted with permission from [38]. Copyright 2010 IOP Publishing.

It is well established that the titania nanotube growth rate is directly proportional to the duration of anodization [3,41,42,43,44,45], the concentration of fluoride ions [3,4,43,45,46], the voltage (Figure 5) [3,43,44,45,46,47], and the electrolyte conductivity [40,46,48]. Aqueous electrolytes limit the nanotube length to 500 nm for acidic and 2 µm for neutral electrolytes since the rate of Equation (3) is faster in aqueous electrolytes [3]. The longest nanotubes reported, 1 mm, required nine days of anodization at 60 V with 0.5 wt % NH_4_F and 3% water in ethylene glycol [37]. With the same electrolyte concentration and voltage, 5 µm long nanotubes are obtained after 17 h [46].

Nanotube formation requiring long anodization durations would not be time-efficient on an industrial scale and several routes have addressed the concern and achieved fast growth rates. Most notably, nanotubes 7 μm long have been grown in 15 s by the addition of 1.5 M lactic acid to the electrolyte solution (Table 2) [36]. Addition of Na_2_EDTA, H_2_O_2_, and up to 0.5 M NH_4_F have been used to increase the nanotube growth rate [31,38,39]. Lactic acid and EDTA assist fluoride by chelating Ti^4+^ and H_2_O_2_ provides an alternate source for oxygen, possibly through radical species [31,36,39]. Fast nanotube growth rates are also determined by a balance between water and fluoride concentration. High fluoride concentration (>0.1 M NH_4_F) enhances the dissolution of TiO_2_ by Equation (3) while addition of water allows for a sufficient rate of titanium oxidation by Equation (1). However, addition of water also slows the dissolution of titania by Equation (3) and is supported by experimental results. Upon addition of 1% water to 0.5 M NH_4_F in anhydrous ethylene glycol, the growth rate increased from 83 nm/min to 308 nm/min at 60 V but 2% water only increased the formation rate to 217 nm/min [43].

**Table 2:** Experimental conditions to achieve efficient lengths.

Length (µm)	Duration	Electrolyte	Voltage (V)	Reference
20	2 h	0.09 M NH_4_F ethylene glycol	60	[48] (SI)
20	0.5 h	0.5 M NH_4_F, 0.25 M Na_2_EDTA, 5% water, ethylene glycol	80	[31]
18	1 min	0.1 M NH_4_F, 1.5 M lactic acid, 5% water, ethylene glycol (60 °C)	150	[36]

**Figure 6:**
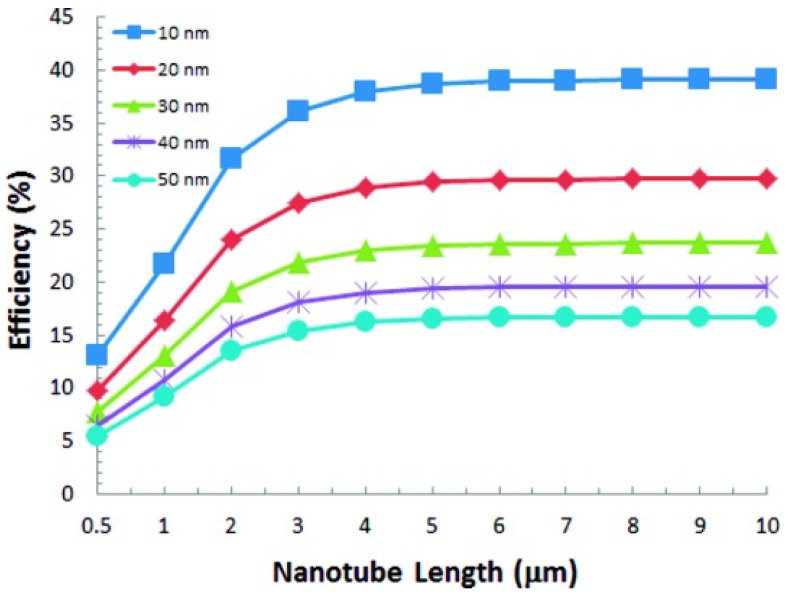
Theoretically efficient nanotube lengths for the reduction of oxygen. The theoretical model considers un-sensitized nanotubes illuminated by UV light. The series corresponds to the inner radius of the nanotubes, ranging from 10 nm to 50 nm. Reprinted with permission from [49]. Copyright 2012 American Chemical Society.

Liu *et al.* used a theoretical model, based on the reduction of oxygen, to determine the most efficient dimensions of un-sensitized titania nanotubes for photocatalysis [49]. Based on the diffusion of oxygen and the molar absorptivity of TiO_2_, the photocatalytic efficiency plateaus with nanotubes greater than 5 µm long (Figure 6) [49]. Experimental results show similar saturation behavior, albeit at longer nanotube lengths [50,51,52,53]. For example, un-sensitized nanotube arrays 12 µm long were most efficient for the degradation of gaseous benzene and toluene when compared to nanotubes ranging from 800 nm to 12 µm in length [50]. Similarly, for the catalysis of acetaldehyde and phenol, the efficiency continued to increase with nanotube length ranging from 200 nm to 17 µm [51,52].

In solar cells using titania nanotubes as the photoanode, there is a balance between absorbing the most photons and reducing the distance the electron must travel in the nanotubes. In accordance with the Beer-Lambert law, more photons are harvested with longer nanotubes that can adsorb more dye or polymer (Figure 4). However, longer nanotubes have more recombination centers, higher series resistance, and lower open circuit potentials [54,55]. Thus, to ensure efficient electron collection, optimized nanotubes lengths do not exceed the electron diffusion length estimated to be 10–100 µm in titania nanotubes and 10 µm in nanoparticles [55,56,57,58].

**Figure 7:**
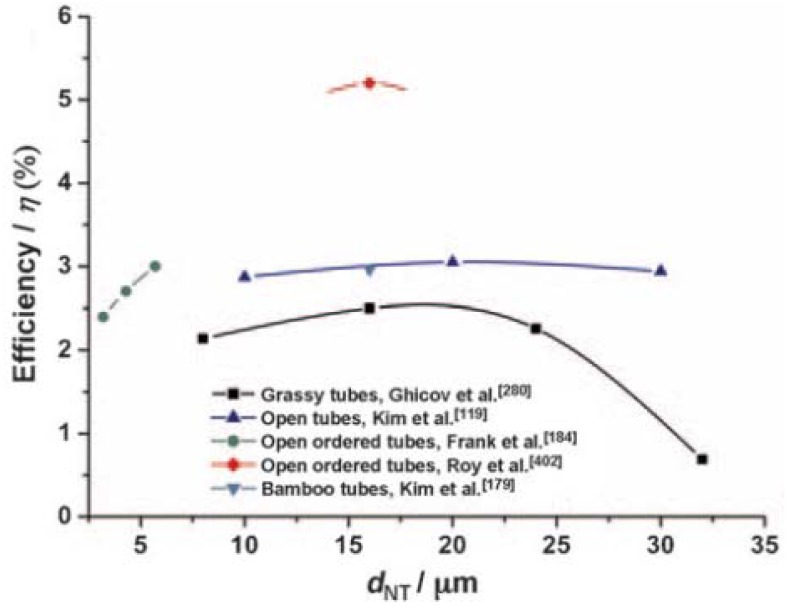
Effect of the length of nanotubes in dye-sensitized solar cells (DSSCs) on the solar power conversion efficiency. Reprinted with permission from [3].

Experimental results demonstrate the nanotube length optimization since the efficiency of hybrid solar cells employing nanotubes decreases after a certain length is exceeded (Figure 7). Park *et al.* found that the photocurrent density increased with increasing TiO_2_ nanotube length up to 35 µm and attributed the effect to the higher surface area for dye-loading [59] Dubey *et al.* found that the photocurrent density and energy conversion efficiency was a maximum for 22 µm long nanotubes (16.3 mA/cm^2^, 6.12%) and decreased at 38 µm because of increased recombination at surface defects [60].

#### 2.1.2. Diameter and Wall Thickness

**Figure 8:**
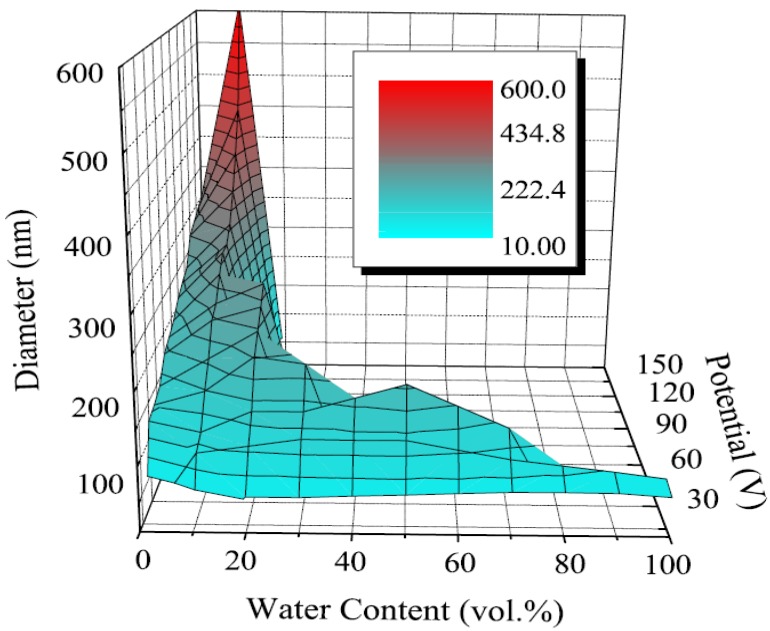
Effect of water content and voltage on the nanotube diameter. Water content under 20% increases the nanotube diameter while limiting the voltage at which nanotubes are formed. Reprinted with permission from [38]. Copyright 2010 IOP Publishing.

Although great control over the nanotube length has been demonstrated, primarily adjusted by the anodization duration, less systematic control over the nanotube diameter and wall thickness has been shown. The nanotube diameter mostly varies with the anodization voltage (Figure 8) [3,29,41,44,45,46,61], but also varies with the solvent [29,32,62], duration [41,46], the water content [38] and the fluoride concentration [11,45]. Nanotubes have been synthesized by anodization that range from 15 nm to 709 nm by adjusting the voltage, solvent, and duration as seen in Table 3 [32,62].

**Table 3:** Experimental conditions to achieve nanotubes with different inner diameters.

Inner Diameter	Duration	Electrolyte	Voltage	Reference
15 nm	24 h	0.2 M HF, 3.6% water, ethylene glycol	10 V	[32]
709 nm	47 h	0.25% HF, 1% water, diethylene glycol	120 V	[62]
80 nm	1.5 h	0.15 M NH_4_F, 3% water, glycerol	80 V	[29]

**Figure 9:**
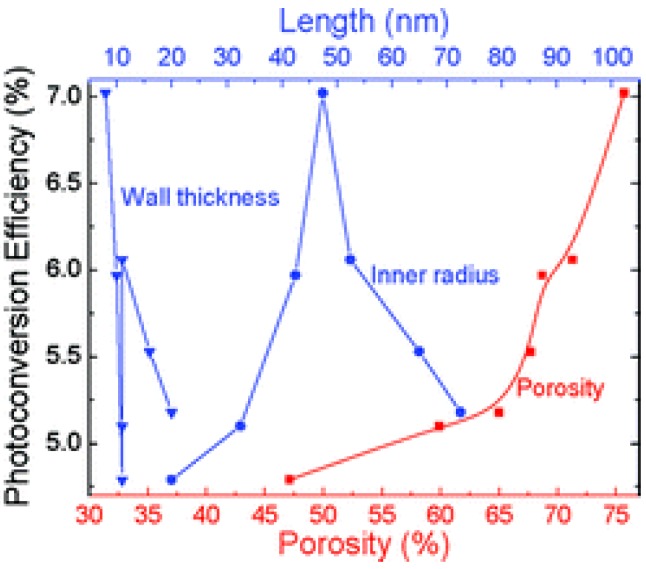
Experimental effect of the porosity (determined by the wall thickness and diameter) on the photoconversion efficiency of water-splitting cells. Reprinted with permission from [63]. Copyright 2012 American Chemical Society.

In addition to the nanotube length, the surface area and efficiency of a nanotube array is determined by the relationship between its diameter and wall thickness (Figure 9) [50,63]. Kontos *et al.* calculated the porosity of their nanotube arrays and compared it to the surface area per unit volume. It is calculated by,

(4) $$P=1-\;\frac{2\pi W(W+D)}{\sqrt{3}{\left(2W+D\right)}^{2}}$$

where *W* is the wall thickness and *D* is the inner diameter. Based the theoretical model used by Liu *et al.*, 20 nm is the most efficient inside diameter for photocatalysis of gaseous reactants (Figure 6) and 20–30 nm, is the most efficient wall thickness [49].

In hybrid solar cells, the high surface area for contact between the electron donor and acceptor (titania) reduces the distance excitons must travel before electrons are collected at the donor-acceptor interface. Thus, excitons are less likely to decay and a higher incident-photon-to-current efficiency (IPCE) is expected, compared to planar solar cells of the same thickness. Exciton diffusion lengths for commonly used organic polymer sensitizers are 8–20 nm for poly(3-hexylthiophene) (P3HT) [22], 20 nm for the poly(p-phenylene vinylene) derivative MEH-PPV [24], and 14 nm for ladder-type poly(p-phenylene) [64].

**Figure 10:**
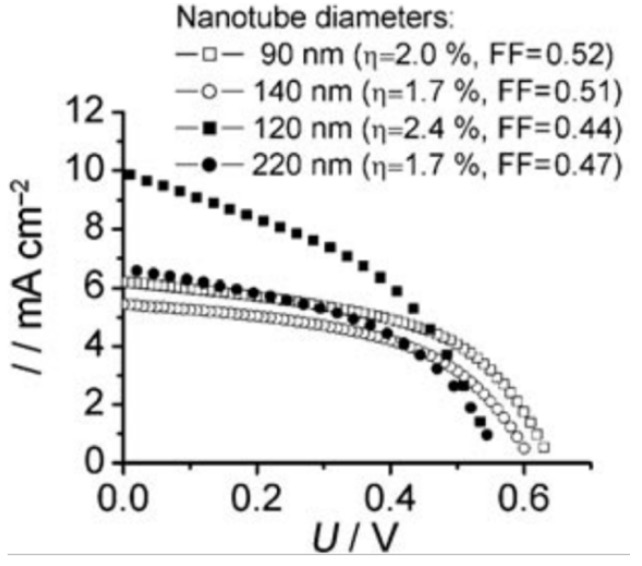
Current-voltage characteristics of DSSCs based on different diameter nanotubes (empty symbols correspond to 8 µm and filled symbols to 16 µm nanotube length). Reprinted with permission from [54]. Copyright 2009 Wiley.

Correspondingly, Ghicov *et al.* found that the nanotube diameter in DSSCs correlated with the solar cell’s short circuit current and photoconversion efficiency, which is attributed to higher dye-loading due to higher surface area (Figure 10) [54]. However, for polymer sensitizers, small diameter nanotubes that have high porosity present an issue for polymer packing within the nanotubes. In the bulk, exciton mobility is enhanced by π-π stacking which allows excitons to delocalize over multiple polymer chains [65]. However, in confining nanotubes, disordered configurations are favored and π-π stacking is largely prevented [65]. Although So *et al.* report that changing the nanotube diameter within 100–200 nm has no significant effect on the solar cell efficiency, 100 nm diameter nanotubes are more efficient at certain lengths (Figure 10) [36,54].

#### 2.1.3. Nanotube Roughness and Intertube Spacing

Titania nanotubes with smooth walls are grown using viscous solvents for anodization. Solvents with high viscosity (Table 4) reduce the mobility of fluoride ions and other ionic species reducing the growth rate, but also reducing current fluctuations and therefore forming smoother nanotube walls [29,34,43,66]. Diffusion through a fluid is inversely proportional to the viscosity of the fluid according to the Stokes-Einstein equation:

(5) $$D=\;\frac{{\text{k}}_{\text{B}}T}{6\pi \eta r}$$

where *D* is the diffusion coefficient of a particle with radius *r*, in a fluid with viscosity *η*, at temperature *T*, and k_B_ is the Boltzmann constant [14,29]. Current fluctuations during the anodization process result from local inhomogenieties of the concentration of ionic species, which cause rough nanotube walls to form [67,68].

**Table 4:** Solvent viscosities.

Electrolyte Solvent	Viscosity (cP) [69]
Glycerol	934
Ethylene Glycol	16.1
Formamide	3.34
DMSO	1.99
Water	0.89

Spacing between nanotubes, on the order of 100 nm, has been achieved by increasing the fluoride concentration and using diethylene glycol. Nanotube arrays grown in diethylene glycol electrolytes have intertube space but require 48 h of anodization to reach 7–20 μm in length [34]. Nanotubes spaced almost 1 µm apart have also been obtained by increasing the HF concentration to 4 wt % in ethylene glycol [70].

Well-ordered nanotube arrays with smooth walls and no intertube contact enhance electron transport by directing the injected electrons toward the conducting substrate and preventing undesired lateral transport between nanotubes [71]. Also, dyes or polymers can be adsorbed to the outside of the nanotubes if they are spaced apart, fully utilizing the available surface area. Intertube spacing smaller than that currently obtained (100 nm to 1 μm) should be more efficient by maximizing the number of nanotubes per unit area. However, no studies on the solar cell or photocatalytic efficiencies of nanotube arrays with intertube space have been published.

### 2.2. Control of Crystallinity

Amorphous titania nanotubes have minimal use in solar energy applications due to the high concentration of recombination centers (Table 5, “unannealed”) [11,44,72]. The anatase crystalline phase of titania is favored due to its higher electron mobility and larger surface area compared to the rutile phase [1,10,73,74]. Although titania nanotubes transformed to mostly rutile while annealing them at 750 °C, the nanotubes collapse at that temperature preventing the experimental comparison between pure rutile and pure anatase nanotubes [44]. Rather, amorphous titania nanotubes are typically annealed at 450 °C in various atmospheres to form the anatase phase [55]. By annealing the nanotubes at temperatures between 450 °C and 750 °C, a mixture of anatase and rutile is formed [44].

The annealing atmosphere affects the anatase-to-rutile phase transformation, and oxygen vacancies and other defects referred to as Ti^+3^ states, which lead to different recombination mechanisms and kinetics [10,11,75]. Ti^+3^ states create an impurity band in the titania nanotubes and limit electron transport, but the number of Ti^+3^ states can be reduced by annealing the nanotubes in an oxygen rich atmosphere [76]. Dry atmospheres inhibit the transformation of anatase to rutile in the nanotube walls while the interfacial region between the nanotubes and the Ti foil substrate transforms to rutile even at 430–450 °C, which may give the false indication that rutile is present throughout the nanotube array [10,11].

Although uncollapsed pure rutile nanotubes have not been studied, mixed phase nanotubes have been shown to be more efficient for photocatalysis than pure anatase nanotubes. The photocatalytic degradation of methyl orange using titania nanotubes is enhanced with rutile/anatase mixing by annealing the nanotube array at 550 °C [77]. Likewise, photodegredation of toluene and rhodamine B by titania powder and nanofibers, respectively, is enhanced when both rutile and anatase are present (3/97 wt %) by using calcination temperatures ≥600 °C [78,79]. Water oxidation is also most efficient after annealing nanotubes at 580 °C where both phases are present [80].

**Table 5:** Data from [44] on the photoconversion efficiency of un-sensitized titania nanotubes annealed at different temperatures in an oxygen atmosphere and illuminated under UV light. All of the samples were fabricated at 30 V for 3 h in 0.27 M NH_4_F, 50 vol % glycerol in water.

Annealing Temperature (°C)	unannealed	350	450	550	650	750
Anatase/Rutile Mass Fraction	amorphous	100/0	100/0	1/1.2	1/2.2	1/37.2
Photoconversion Efficiency (%)	1.4	5.86	5.93	7.25	8.56	0.4

**Figure 11:**
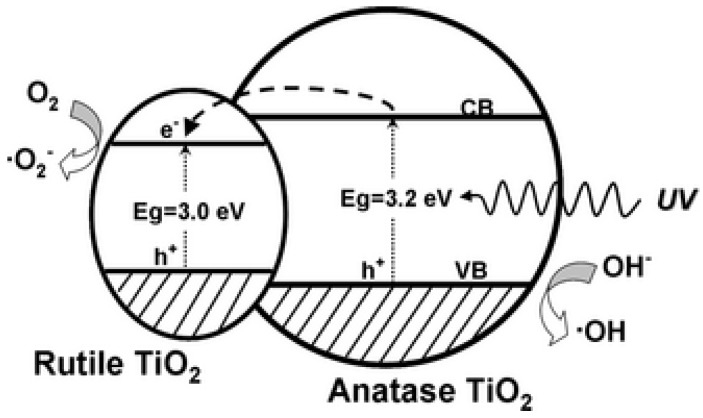
Proposed schematic illustration of the band structure related to photocatalytic mechanism of un-sensitized mixed-phase TiO_2_ structure. Reprinted with permission from [78]. Copyright 2011 Wiley.

To explain the mixed-phase phenomenon, Li *et al.* proposed that the rutile crystals provide electron trapping sites that extend the lifetime of photo-generated electron-hole pairs (Figure 11) [78]. However, Richter *et al.* found that rather than increasing the electron lifetime, the calcination temperature reduces the number of exciton-like trap states from oxygen vacancies, therefore improving electron transport [60]. Ghicov *et al.* attributed the mixed–phase phenomenon to increased crystallization at 600 °C, which minimized the number of recombination centers by reducing the amount of grain boundaries and amorphous TiO_2_ [11].

Although there are many studies on un-sensitized crystalline nanotubes, systematic studies of crystallinity on titania nanotube solar cells is lacking. However, the crystallinity of nanoparticles has been studied. Anatase nanoparticle films have a higher energy conversion efficiency (21%), photocurrent (30%), and electron diffusion length (10×) than rutile nanoparticle films of the same thickness [81]. The difference was partly attributed to the increased interparticle contact and dye loading from the higher surface area of the anatase nanoparticles [81].

## 3. Solar Cell Fabrication

**Figure 12:**
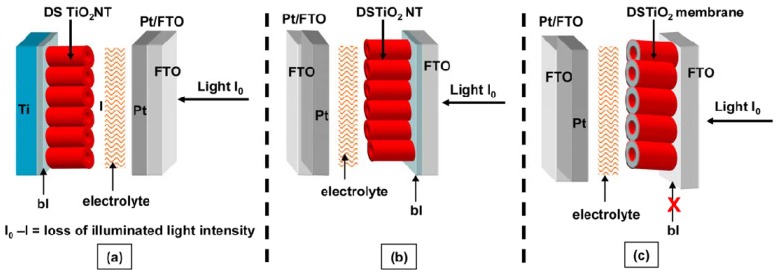
Different orientations of DSSCs with TiO_2_ nanotubes. “bl” represents the barrier layer of TiO_2_ at the closed ends of the nanotubes. (**a**) Backside illumination with nanotubes on titanium foil; (**b**) Front-side illumination with nanotubes on fluorine-doped tin oxide coated glass (FTO glass); (**c**) Frontside illumination with nanotubes open on both ends. Reprinted with permission from [31]. Copyright 2010 IOP Publishing.

Hybrid solar cells benefit from front-side illumination where light is incident on the transparent conducting oxide and immediately reaches the sensitized TiO_2_ nanotube array (Figure 12b,c) [31,48]. In this orientation, reflection and absorption of light by the counter electrode and electrolyte is avoided. Nanotube arrays left on the titanium foil substrate can only be used in the less efficient back-side illuminated solar cell configuration since the foil is opaque (Figure 15a) [31,48]. Two routes have been used to fabricate front-side illuminated solar cells with TiO_2_ nanotube arrays: (1) transferring the nanotube array from the titanium foil to fluorine-doped tin oxide coated glass (FTO glass) [48,59] and (2) anodizing a film of titanium sputter-coated onto FTO glass [29].

### 3.1. Removing the Array

**Figure 13:**
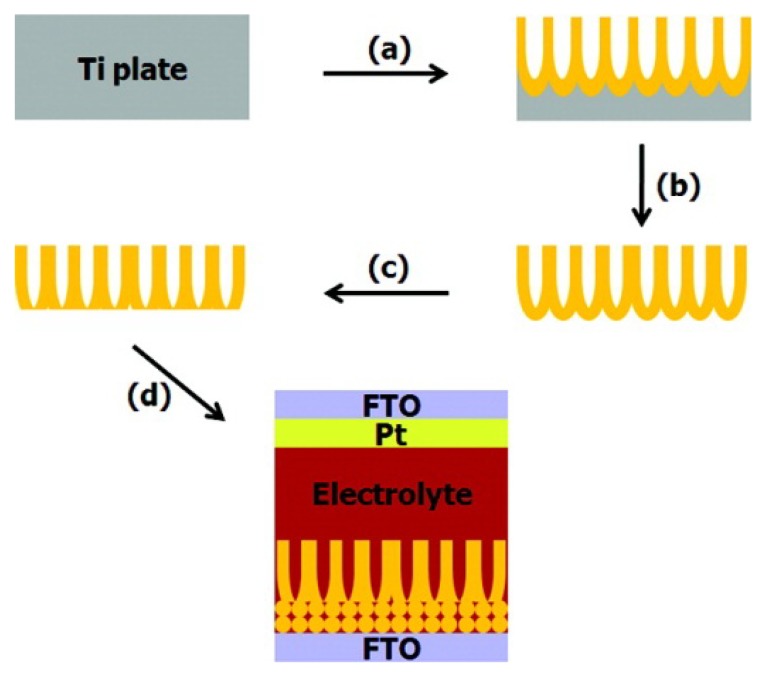
Scheme illustrating the transferal of a nanotube array from titanium foil to FTO glass coated with TiO_2_ nanoparticles for adhesion. Process (**a**) is the anodization; (**b**) the nanotube removal; (**c**) the removal of the barrier layer; and (**d**) the adhesion to FTO glass. Reprinted with permission from [87]. Copyright 2012 American Chemical Society.

Titania nanotube arrays have been removed from the Ti by dissolution in a bromine/methanol solution [82], aqueous HCl [59,83], solvent-evaporation of methanol [84], ultrasonication in water [46], acetone [85], ethanol/water solutions [86], and drying in air [31]. After removing the nanotube array, it can be attached to FTO glass with a few drops of 100 mM titanium isopropoxide or a layer of TiO_2_ nanoparticle paste 3 µm thick and then annealed (Figure 13d) [31,59,60,78]. Dubey *et al.* enhanced the adhesion by putting a 100 g weight onto the nanotube-FTO glass assembly in a freezer [60].

### 3.2. Anodizing on Conductive Substrates

Titania nanotubes have been grown by anodization of titanium films sputter-coated on alumina, indium-doped tin oxide [88] coated polyethylene terephthalate (ITO PET) [29], fluorine-doped tin oxide coated glass [29,48]. Titania films 0.5 to 20 µm thick have been coated onto the conducting substrates by RF or DC magnetron sputtering and subsequently anodized in electrolytes containing fluoride to form nanotubes [29]. To improve the adhesion of titanium films to FTO glass, the glass is heated to 45–400 °C before deposition [29,48] and bombarded with Ar^+^ during deposition of the titanium films [48]. By bombarding the titanium film with ions during deposition, weakly bound titanium atoms are removed, leaving the titanium atoms strongly bound to the substrate.

### 3.3. Removal of Barrier Layer

The closed ends of the nanotubes (barrier layer), originally attached to the titanium foil hinder light absorption from front-side illuminated hybrid solar cells [31,87]. Although nanotubes grown directly on conductive substrates suffer from the light reflection by the barrier layer, the barrier layer can be removed from nanotube arrays transferred from titanium foil. After removing the nanotube array from the titanium foil, the barrier layer can be removed by HF etching [31] or ion milling [87], similar to ion milling carbon nanotubes [89]. In the ion milling technique, the barrier layer is bombarded with Ar^+^ and removed by the sputtering process.

**Figure 14:**
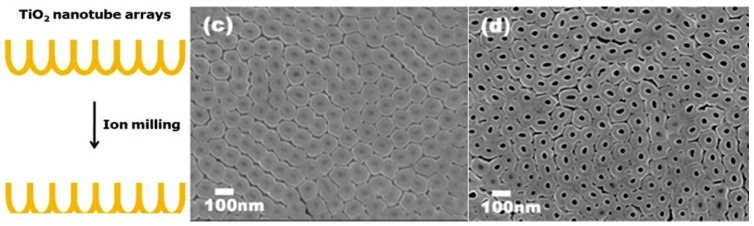
SEM images of the closed-end/backside of TiO_2_ nanotube arrays removed from the Ti foil after (**c**) 30 min and (**d**) 90 min of ion milling. Reprinted with permission from [87]. Copyright 2012 American Chemical Society.

**Figure 15:**
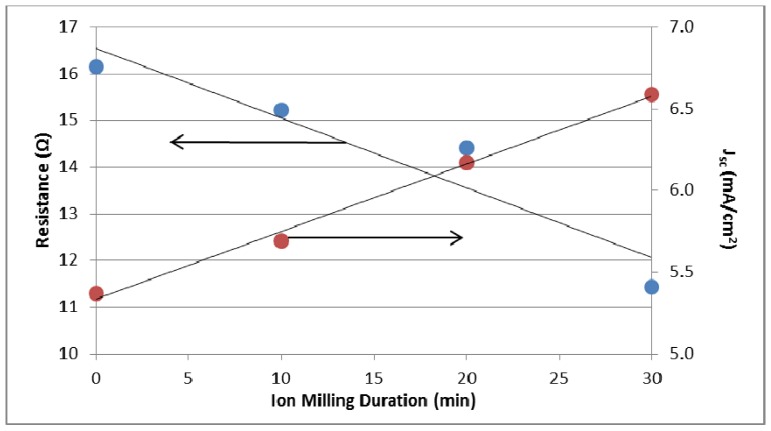
Effect of argon ion milling duration on the nanotube transport resistance and short circuit current measured from electrochemical impedance spectra. The nanotubes were sensitized with N-719 dye and illuminated with AM 1.5 illumination. Data from [87]. Copyright 2012 American Chemical Society.

The barrier layer thickness decreases with increasing argon ion milling duration (0–90 min) and the barrier layer is perforated after 90 min as seen in Figure 14d [87]. Under backside-illumination of the ion milled N-719 sensitized solar cell, the photocurrent and energy conversion efficiency increased by 46% and 48% to 7.85 mA/cm^2^ and 3.7% , respectively, after 90 min of ion milling the nanotubes [87]. From electrochemical impedance spectroscopy measurements, Rho *et al.* determined that the barrier layer contributes to transport resistance in the nanotubes (Figure 15) [87]. Since the open-circuit voltage, V_oc_, is dependent on the recombination rate and there is no significant difference among the V_oc_ for different barrier thicknesses, only the electron transport rate is affected [87].

As an alternative to removing the barrier layer, Dubey *et al.* configured solar cells with the nanotube opening facing the FTO glass, effectively removing the barrier layer from the nanotube-FTO glass interface [60]. This configuration led to better contact with the FTO layer, higher photoconversion efficiencies (*η* = 6.1% *vs.* 3.8%), and open-circuit current densities attributed to reduced recombination from better contact and twice the dye loading [60]. Dubey *et al.* achieved higher efficiencies with their inverse nanotube configuration than did Rho *et al.* with their ion milling technique (6.1% *vs.* 3.7%) using the same dye (N-719) and similar nanotube lengths (22 µm *vs.* 18 µm) [87]. The adhesion of the inverse nanotube configuration to the FTO glass may be superior to the ion milled nanotubes since Dubey *et al.* applied pressure and cold temperatures as described in Section 3.1.

## 4. Conclusions

The extensive research on anodized titania nanotube arrays has led to steady improvements of its morphological control [3,25]. A wide range of nanotube array dimensions have been grown and tested in various solar energy conversion applications for optimum performance (Table 1). Great advances have been made in controlling the nanotube length since the first anodized titania nanotube report, but systematically controlling the nanotube diameter and wall thickness to the narrow dimensions that are theoretically efficient requires continued research. Considering that the titania nanotubes’ crystallinity drastically affects its photoconversion efficiency and electron dynamics, the electron behavior in mixed-phase nanotubes requires attention to resolve disagreement in the literature [11,76,78]. Studies on hybrid solar cells with mixed-phase titania nanotubes may contribute to the understanding of the mixed-phase phenomenon in un-sensitized nanotubes.

Further optimization and characterization of the attachment of nanotube arrays to conductive substrates could benefit electron transport by reducing transport resistance between the phases [29,41,60]. For BHJs, improving polymer π-π packing and preventing phase separation with titania is needed to fully utilize the surface area available in the nanotubes and enhance photoconversion efficiencies [21].
